# Integrating microbial communities into algal biotechnology: a pathway to enhanced commercialization

**DOI:** 10.3389/fmicb.2025.1555579

**Published:** 2025-04-01

**Authors:** Hari Koneru, Safiatou Bamba, Aksel Bell, Adrian A. Estrada-Graf, Zackary I. Johnson

**Affiliations:** ^1^Marine Laboratory, Nicholas School of the Environment, Duke University, Beaufort, NC, United States; ^2^Biology, Civil and Environmental Engineering, Duke Microbiome Center, Duke University, Durham, NC, United States

**Keywords:** marine, algae, microbiome, biotechnology, interactions

## Abstract

Microalgae are increasingly recognized for their potential in wastewater treatment and the sustainable production of feedstock for fuel, feed, food, and other bioproducts. Like conventional agricultural systems, algal cultivation involves complex microbial communities. However, despite their pivotal role in cultivation outcomes, especially at the commodity-scale, the critical interactions between microalgae and their microbiomes are often overlooked. Here we synthesize current knowledge on the taxonomic diversity, ecological roles, and biotechnological potential of algal microbiomes, with a focus on their interactions with algal hosts through nutrient exchange, growth modulation, pathogen defense, and environmental conditioning. We also examine how environmental factors such as nutrient availability, salinity, and temperature influence these interactions. Advances in microbiome engineering, including synthetic biology and ecological approaches, offer opportunities to enhance beneficial algal-microbiome interactions, thereby improving growth, resilience, and yield. These advancements could lead to more sustainable and economically viable microalgae cultivation, with far-reaching implications for environmental management and biotechnological innovation. By addressing key economic and environmental barriers, microbiome engineering holds transformative potential to revolutionize large-scale algae cultivation and provide sustainable solutions to global challenges.

## 1 Introduction

There is an increasing urgency to develop sustainable approaches to address the rising demand for resources amidst changing environments. Microalgae are recognized as some of the most efficient biological agents for CO_2_ fixation (Barati et al., 2022), offering significant potential for reducing the carbon footprint of industrial processes and moving closer to achieving carbon neutrality. Because of their vast genetic, biochemical and physiological diversity microalgae have shown promise for a variety of applications including wastewater treatment, and the sustainable production of feedstock for fuel, feed, food, and other bioproducts (Greene et al., 2022).

Despite its promise, large-scale commercialization of microalgae cultivation has faced several challenges including high capital equipment and operational costs as well as unrealized consistent high algal productivities (Beal et al., 2015; White and Ryan, 2015). Novel approaches such as genetic engineering of algae and improved bioreactor design promise to boost algal productivity and cut equipment costs, while automation and larger functional units allow for scalable algae cultivation (Novoveská et al., 2023). In addition to these improvements, one often overlooked factor is the microalgae-associated microbiomes: diverse communities of bacteria, fungi, and other microorganisms that dramatically influence algae production. These microbiomes can have both beneficial and detrimental effects on algal hosts, making them a key area of interest for advancing algal-based biotechnologies. While impractical at commodity scale, axenic microalgae cultivation often results in phenotypic changes and reduced growth rates (Lian et al., 2018) bluntly demonstrating the importance microbiomes. Microbiomes can enable an increase in growth rates, robustness to environmental conditions, harvesting efficiency, and resistance to pathogens, all of which are key barriers to large scale algae cultivation. Thus, by leveraging microbiome interactions to enhance realized algal productivity, we can develop more effective strategies for large-scale production of algae-derived bioproducts.

This minireview aims to synthesize current knowledge on algae-associated microbiomes with a focus on their biotechnological applications. We highlight novel strategies for microbiome optimization, including bacteriophage-based modulation, AI-driven design, microbiome transplantation, and synthetic microbiome engineering. These insights may lead to microbiome optimization toward enhancing microalgae as a sustainable solution to address the increasing global demands for fuel, food, and other bioproducts.

## 2 Algal-microbiome interactions

Algae and bacteria engage in diverse ecological interactions that influence algal growth and productivity (Cole, 1982). These interactions range from mutualism to parasitism (Ramanan et al., 2016) and play a critical role in commercial algae cultivation (Lian et al., 2018). For example, *Chlorella ellipsoidea* benefits from *Brevundimonas*, increasing growth up to threefold (Park et al., 2008), whereas antagonistic bacteria like *Pseudomonas protegens* produce algicidal toxins that inhibit algal growth (Aiyar et al., 2017; Rose et al., 2021). These relationships impact algal productivity through growth modulation, nutrient exchange, pathogen interactions, and environmental conditioning.

### 2.1 Growth modulation and nutrient exchange

Bacteria can enhance algal growth by producing auxins and growth-promoting hormones (Amin et al., 2015; Berthold et al., 2019), while algae can regulate their microbiome through antimicrobial compounds (Desbois et al., 2009). However, some bacteria negatively impact algal growth by outcompeting them for nutrients or producing toxins (Coyne et al., 2022). Nutrient cycling, including nitrogen fixation and phosphate solubilization, is often central to algae-microbiome interactions (Foster et al., 2011; Wienhausen et al., 2017). Vitamins, iron, dissolved carbon, and nitrogen can be the currency of many algae-bacteria interactions (Amin et al., 2012; Gujar et al., 2025) and bacterial strains can improve algal health by increasing the bioavailability of these and other nutrients (Wienhausen et al., 2017; Amin et al., 2009; Ashraf et al., 2023). Specific bacteria strains promote lipid synthesis and nitrogen availability through other mechanisms (Berthold et al., 2019; Liu B. et al., 2020). The *Roseobacter* clade often correlates with microalgae population density across a broad range of environments, and they have important effects on the environment by cycling elements like carbon and sulfur via oxidization of carbon monoxide and production of dimethylsulfide (Geng and Belas, 2010). In some cases, both microalgae and bacteria growth can be limited by the same nutrients such as nitrogen, phosphorous, and iron. In other cases, some members of the microbiome can outcompete their hosts for resources because of their high surface to volume ratio (Guerrini et al., 1998). Such competitive effects can occur across a range of nutrients, and result in reduced growth rates for algal hosts and their microbiome.

### 2.2 Pathogens and environmental acclimation

The microbiome can protect algae from pathogens through biofilm formation and secretion of extracellular polymeric substances (Saha and Weinberger, 2019). Certain bacteria also confer immunity against algal viruses (Kimura and Tomaru, 2014). Conversely, some bacteria exhibit algicidal properties that cause cell lysis (Demuez et al., 2015; Hotter et al., 2021). *Phaeobacter inhibens* triggers the programmed cell death of its algal host *Emiliania huxleyi*, leading to the collapse of large algal blooms (Bramucci and Case, 2019). The *Bdellovibrio* strain FD111 was found to cause algal culture loss in outdoor ponds (Lee et al., 2018). *Vampirovibrio chlorellavorus* is a parasitic bacterium that latches onto its *Chlorella* hosts and can crash industrial algae cultures (Ganuza et al., 2016). *Bacillus safensis* secretes growth-inhibiting molecules into algal cultures, resulting in low productivity (Fulbright et al., 2016). Microbiomes enhance algal tolerance to environmental stressors, such as salinity fluctuations, oxidative stress, and nutrient limitation (Dittami et al., 2016; Wang T. et al., 2024; Morris et al., 2008). For instance, microbes mitigate stress by reducing reactive oxygen species and increasing exopolysaccharide production (Xiao et al., 2022; Morris et al., 2011). Changes in nutrient availability can modulate these algae-bacteria interactions. Nitrogen and phosphorus ratios can shift relationships from mutualistic to competitive (Fuentes et al., 2016; Zhang et al., 2021).

## 3 Factors influencing changes in microbiome composition

Microbiome structure is shaped by host species, environmental conditions, and cultivation scale, and starting composition among other factors. Closely related microalgae harbor distinct bacterial communities, which are phylogenetically distinct from free-living bacteria (Grossart et al., 2005; Cirri and Pohnert, 2019; Ahern et al., 2021; Steinrücken et al., 2023). While algae strain is a major determinant of microbiome composition, environmental factors such as nitrogen availability, salinity, temperature, and pH also play a role (Kimbrel et al., 2019; Xiao et al., 2022; Biondi et al., 2017; González-Camejo et al., 2019). For example, high salinity can increase bacterial abundance but also trigger algal population crashes (Saha and Weinberger, 2019). In large-scale outdoor cultivation, microbiome diversity shifts with scale; in *Nannochloropsis* cultures, Bacteroidetes and Proteobacteria dominate, but their relative abundance changes as cultivation expands from laboratory to open-pond systems (Liu B. et al., 2020; Fulbright et al., 2018).

Understanding the composition and ecological roles of algae-associated microbiomes is essential for harnessing their potential in biotechnological applications. Rather than viewing microbiomes as passive environmental factors, they should be considered key components in optimizing algal productivity and commercial feasibility.

## 4 Biotechnological applications of optimized microbiomes

**Figure 1:**
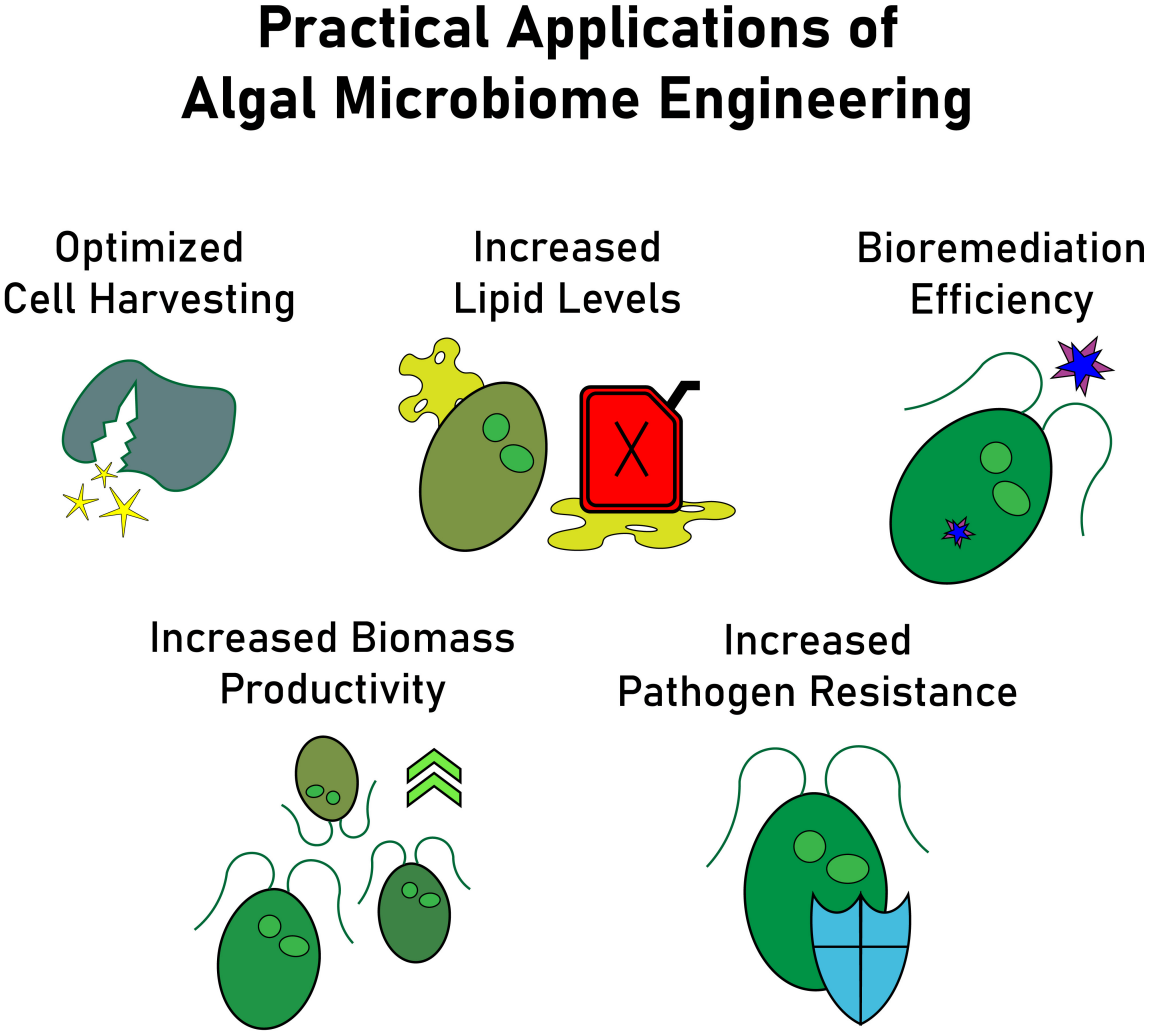
Applications of microbiome engineering across the industrial microalgae cultivation pipeline, enhancing biomass productivity (Berthold et al., 2019), increasing lipid levels for biofuel production (Chorazyczewski et al., 2021), improving bioremediation efficiency for contaminant and heavy metal removal (Okurowska et al., 2021; Greeshma et al., 2022), strengthening pathogen resistance, and optimizing cell harvesting through microbial bioflocculation (Fuentes et al., 2016).

As interest in large scale algae culture grows, so has the recognition of the importance of microbiomes in optimizing realized algal productivity (Fuentes et al., 2016). By purposely and deliberately integrating bacteria into the algae production pipeline, costs associated with synthetic chemicals, nutrients, energy, harvesting, and product recovery could be significantly reduced. Microbiome engineering also has the potential to enhance biomass production, specific compounds such as lipids, bioremediation efficiency, and pathogen resistance (Figure 1). For instance, some bacterial species stimulate both biomass and lipid concentration in algae, a promising result for biofuel production (Berthold et al., 2019). Similarly, some bacteria correlate with increased lipid production, while others influence extracellular metabolites relevant for large-scale algal growth (Chorazyczewski et al., 2021). Beyond biofuel applications, algae-based bioremediation of landfill leachate was improved by microbiome enhanced contaminant removal (Okurowska et al., 2021). Similarly, microbiomes can boost algae's ability to remove heavy metals, demonstrating the potential for wastewater treatment applications (Greeshma et al., 2022). Microbiome engineering also facilitates novel approaches to algae harvesting and product recovery such as using bioflocculation mediated by microbes to streamline harvesting processes (Fuentes et al., 2016). Additionally, microbial signals may be harnessed to lyse algal cells, preserving high intracellular product yields.

These applications underscore the critical role of microbiomes in algal biotechnology. Far from being passive components of cultivation systems, microbiomes can actively enhance productivity, stability, and product quality, reinforcing the need for targeted microbiome management strategies.

## 5 Methods for optimization of algal microbiomes

**Figure 2:**
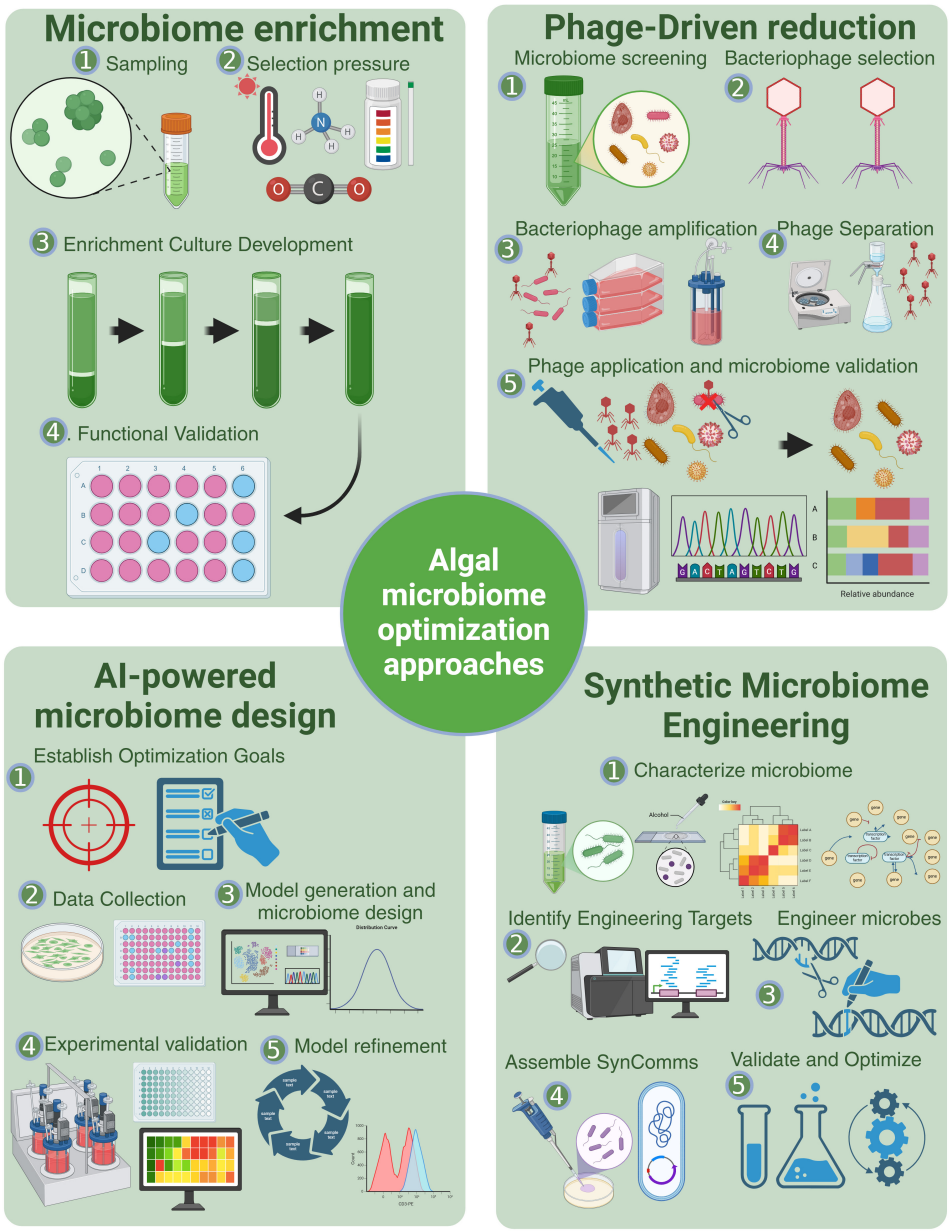
Methods for optimization of algae microbiomes. Microbiome enrichment applies selection pressures to cultivate beneficial microbial consortia, an approach commonly used across diverse microbiome types (Brugman et al., 2018). Phage-driven reduction leverages bacteriophages to selectively suppress undesirable microbial taxa, allowing for microbiome refinement (Foo et al., 2017; Tanaka et al., 2024). Synthetic microbiome engineering integrates genome characterization, microbial engineering, and synthetic consortia assembly to construct functionally optimized microbiomes (Ke et al., 2021). AI-powered microbiome design employs machine learning models to predict microbiome structure and function based on environmental and metabolic data, as demonstrated by Liu et al. (2023) and Kessell et al. (2020).

Motivated by potential improvements to the industrial algae cultivation pipeline, the rational design of algal microbiomes is being pursued using a variety of traditional and modern strategies for optimization (Figure 2). These approaches can be classified as “top-down” and “bottom-up” approaches (Sorbara and Pamer, 2022), with each having advantages and challenges.

The simplest, but perhaps most indirect “top-down” method modulates the microbiome by altering the algae's environment. Mixed microbial communities can be subject to varying environmental conditions and the metabolic profile of generated microbial consortia can then be measured. This host-mediated microbiome engineering approach has been demonstrated in traditional agricultural systems such as tomato plants that when grown under water-deficient conditions over generations of repeated host selection, the microbiome cultivated by the host significantly increased tomato seedling tolerance against water deficit stress (Rodríguez et al., 2023). Other traditional enrichment-based methods for top-down modulation of microbial community composition, including prebiotics, antagonistic probiotics, and microbiome transfer, have been applied across diverse microbiome types (Brugman et al., 2018). For plants including algae, prebiotics can include adding nutrients that favor specific microbes to guide the community structure of the microbiome. Microbiome transfer involves transplanting microbial-rich media, often derived from environments optimized for another host. Synthetic biology advances have additionally allowed for high-throughput top-down methods such as broad viral transfections and engineered mobile genetic elements. For example, an *E. coli* donor strain has been used to deliver a mobile genetic element to a microbiome via bacterial conjugation for broad-range metagenome engineering (Ronda et al., 2019). Such methods can flexibly allow the insertion of genes into microbial genomes that benefit algal productivity and growth (Ke et al., 2021).

Bottom-up approaches isolate individual species and assemble a predicted symbiotic (and potentially synergistic) community. Beneficial probiotics, a common strategy in algal microbiome studies, also align with this approach by introducing favorable bacteria directly, such as through the seeding of culture media with a single strain or multimember communities. Advances in synthetic biological tools are transforming how algal microbiomes are understood and how they can be designed and engineered. For example, a broad range retron-based system to record a timeline of cellular interactions and spatial information into the genome of cells within a microbiome has been developed (Farzadfard et al., 2021). These genomes can subsequently be sequenced to allow for a much more detailed understanding of microbiome dynamics, which may help in the rational design of bottom-up approaches to microbiome engineering. The improved ability to modify non-model organisms via broad host-range plasmids has allowed for the editing of individual species to carry plant growth promoting genes. Bacteriophages can also be valuable tools for targeted microbiome manipulation. Engineered bacteriophages can selectively target and suppress undesirable microbial taxa, enabling the fine-tuning of microbial communities by reducing competition or promoting symbiotic microbes (Foo et al., 2017). These tools, while initially developed for human gut microbiomes, have potential applications in algal systems for improving productivity and managing contamination. For example, virulent bacteriophages can be used to suppress the growth of target species within a microbiome without compromising growth of non-target species (Tanaka et al., 2024). These approaches can facilitate the study of specific bacterial species and their roles in microbiomes, as well as the precise design of microbiomes that exclude algae growth-inhibiting bacteria.

Computational advancements such as novel machine learning methods provide another mechanism to understand and rationally design microbiomes. Training a random forest classifier on microbial community compositions, environmental and hydrological data has been shown to accurately predict microbiome structure (Liu et al., 2023). Similarly, machine learning has been used to predict symbiotic communities of minimal size based on metabolite profiles and to simulate microbiome success (Kessell et al., 2020). Computational tools also help elucidate microbial interactions by analyzing species abundance data to construct interaction networks. A deep learning trained long short-term memory model on human gut microbiome constituents and metabolites could accurately predict microbiome trajectories, identify relevant species and metabolite interactions, and was ultimately used design a synthetic microbiome with health-relevant metabolite profiles (Baranwal et al., 2022). Outside of deep learning tools, similar predictions of human gut microbiome metabolites such as butyrate have been made using community dynamics models and linear regression (Clark et al., 2021). A microbiome modeling framework to simulate metabolic events was used to screen various combinations of keystone bacterial species (Ruan et al., 2024), which lead to an accelerated design process to optimize the biodegradation of harmful herbicides by microbiomes. A variety of such computational tools promise to elucidate the complex interactions within microbiomes and thereby allow for the rational design of microbiomes for a target metabolite profile or host outcome.

With these recent improvements to microbiome engineering tools, investment in microbial solutions for the farming industry has increased. In the agriculture sector, Pivot Bio produces a biofertilizer that uses a mixture of nitrogen fixing bacteria to boost the growth of corn (Woodward et al., 2025). AgBiome leverages computational tools and their data on *Bacillus* genomes to understand soil microbe metabolite profiles (Grubbs et al., 2017). Robigo genetically engineers microbes to act as biological pesticides (Wallace, 2023). The use of more novel methods, alongside traditional methods like microbiome transfers, may similarly allow for commercial algal cultures that grow more efficiently, yield more product, and avoid contamination all at a lower cost. Currently, a lack of widely available data on microbiome function, host specificity, and environmental sensitivity in large-scale cultures slows this effort.

Advancements in microbiome engineering, including synthetic consortia, AI-driven modeling, and targeted microbiome modulation, offer new opportunities to systematically enhance algal growth and stability. These strategies pave the way for precision microbiome optimization in large-scale cultivation.

## 6 Outlook

Microalgae and their microbiomes form dynamic partnerships that play essential roles in natural aquatic ecosystems, but that are also important in industrial cultivation. Natural microalgal systems thrive in association with microbial consortia, which support nutrient cycling, growth, pathogen defense, and environmental resilience. Further, cross system comparisons could provide insight for algal biotechnology. For example, studies on gut microbiomes have revealed how microbial diversity and functional complementarity enhance nutrient assimilation, disease resistance, and host metabolism (Sankararaman et al., 2023; Wang W. et al., 2024; Qi et al., 2023). These findings suggest opportunities to engineer algal microbiomes for more efficient nutrient uptake, improved resilience to pathogens, and enhanced productivity under varying environmental conditions. Similarly, tools for terrestrial agriculture such as biological control agents (BCAs), biofertilizers, and biostimulants, which have proven effective in enhancing plant growth and resilience, offer valuable insights for algal microbiome manipulation (Berg et al., 2021; Du et al., 2021; Liu H. et al., 2020). Algae-based biostimulants and biofertilizers are already being commercialized for agricultural applications. For example, a microalgae-derived biostimulant enhances plant growth and stress tolerance (Fornieles, 2023), algae-based biofertilizers improve soil microbiota and crop productivity (Osorio-Reyes et al., 2023) and seaweed is used as biofertilizers and biostimulants for sustainable agriculture (Nivetha et al., 2024). These commercial applications highlight the potential of microbiological inputs in industrial-scale cultivation systems. However, lessons from agriculture also highlight the challenges of applying reduced microbial consortia, which often struggle to establish dominance over the highly diverse and competitive soil microbiome (Joubert et al., 2024). This underscores the importance of carefully considering the resilience and complexity of natural microbiomes when designing interventions for algae cultivation.

Second-generation microbiome technologies applied to plant sciences offer promising strategies to overcome these obstacles. Microbiome transplantation, *de novo* synthetic community design and application, and microbiome modulation have emerged as tools to address the challenges posed by priority effects and the complexity of wild microbiomes (Compant et al., 2024). These approaches aim to refine microbial composition and functionality, enabling targeted improvements in plant health and productivity. Synthetic biology and machine learning further offer advanced precision, enabling the design of microbiomes tailored to specific industrial objectives. Algal microbiome manipulation can leverage these advances to further develop strategies that enhance establishment success and functionality in diverse cultivation environments.

Machine learning presents new opportunities for optimizing algal microbiomes by leveraging existing datasets to predict microbial interactions and community stability. For example, the mutualistic relationship between *Chlorella ellipsoidea* and *Brevundimonas* (Park et al., 2008), known to enhance algal growth, provides a foundation for training models to identify additional beneficial partnerships. Similarly, the algicidal activity of *Pseudomonas protegens* (Aiyar et al., 2017; Rose et al., 2021) can be incorporated into predictive frameworks to anticipate harmful bacterial blooms and inform mitigation strategies. Models integrating nutrient exchange dynamics, such as nitrogen fixation and phosphate solubilization (Amin et al., 2012; Berthold et al., 2019), could further refine nutrient supplementation protocols to optimize biomass production. Reinforcement learning approaches may also facilitate *in-silico* simulations of microbiome dynamics, allowing for iterative refinement of microbial consortia with targeted functional traits. By integrating microbiome sequencing data with machine learning predictions, future studies can transition toward a data-driven, rational design of algal microbiomes tailored for specific biotechnological applications.

Emerging applications of using microbiomes to enhance algal biomass yields, water treatment, improve product recovery, and mitigate against contamination hold transformative potential. By integrating functional data on microbiome-algae interactions with lessons from both animal and terrestrial systems, we can accelerate progress in bioremediation, biofuel production, and bioproduct harvesting. For example, the use of microbial consortia designed to metabolize waste products into usable nutrients could reduce input costs while increasing efficiency.

To fully harness the potential of microbiome engineering in algal biotechnology, a systematic approach is required. First, identifying target outcomes such as enhanced biomass productivity, lipid accumulation, or stress tolerance is essential. Second, characterizing native algal microbiomes through metagenomics and functional profiling to define beneficial microbial consortia provides critical baseline information (Kimbrel et al., 2019; Cirri and Pohnert, 2019). Third, rational design strategies, including top-down enrichment of naturally co-occurring microbes and bottom-up synthetic assembly of functionally complementary strains, can be used to enable the development of optimized microbiomes (Ke et al., 2021; Foo et al., 2017). Finally, scalable implementation requires controlled experimental validation, followed by pilot-scale testing under commercial conditions to ensure microbial stability and performance (Lian et al., 2018; Fulbright et al., 2018). Real-time monitoring tools and AI-driven predictive modeling can further refine microbiome applications, leading to standardized microbial consortia for large-scale cultivation (Liu et al., 2023; Kessell et al., 2020). By integrating these steps, microbial community engineering can transition from a conceptual roadmap to a practical tool for enhancing the commercialization of algae-based bioproducts.

Despite its potential to address many grand sustainability challenges, algal biotechnology and its microbiome engineering faces challenges due to the incomplete understanding of the molecular to ecological mechanisms underlying algal-microbiome interactions (Lian et al., 2018). Many interactions are species-specific, requiring tailored approaches for each application until a more general framework for bacterial co-culture can be developed. While laboratory experiments are essential for uncovering many of the fundamental mechanisms of algal-microbiome interactions, their findings do not always translate directly to large-scale cultivation (Corcoran et al., 2022), where environmental variability and microbial community dynamics differ significantly. Future research should prioritize validating these interactions in field-scale systems to assess their functional relevance under real-world conditions. As tools and techniques for microbiome engineering mature, they will contribute to the economic and environmental sustainability of large-scale microalgae cultivation. By bridging disciplines and embracing novel technologies, the integration of microbial communities into algal biotechnology can redefine the boundaries of industrial cultivation, driving sustainable, and economically viable solutions for global challenges.
